# 971. Pharmacokinetic Estimation Analysis of Multi-Dose Regimens of Dalbavancin for Treatment of Osteomyelitis or Bacteremia

**DOI:** 10.1093/ofid/ofad500.026

**Published:** 2023-11-27

**Authors:** Cecilia Volk, Paul Hutson, Warren Rose

**Affiliations:** University of Wisconsin-Madison, Madison, Wisconsin; University of Wisconsin- Madison, School of Pharmacy, Madison, Wisconsin; University of Wisconsin-Madison, Madison, Wisconsin

## Abstract

**Background:**

The management of multi-drug resistant gram-positive infections such as methicillin-resistant *Staphylococcus aureus* (MRSA) is a complicated task. This is made more difficult when prolonged treatment durations are required, such as for bacteremia, endocarditis, osteomyelitis, or other deep-seated infections. Dalbavancin (DAL) is a long-acting lipoglycopeptide increasingly being used for these infections and is a favorable option when clinicians desire to avoid central catheter placement. Despite the increasing use of DAL for prolonged therapy, there is a lack of consensus on optimal dosing and pharmacokinetic attainment.

**Methods:**

An *in silico* pharmacokinetic/pharmacodynamic simulation was performed to assess the predicted DAL concentration resulting from either two 1500mg doses separated by one week, or a 1000mg loading dose followed by 5 weekly doses of 500mg. Single dose population PK parameters describing concentrations in serum as well as two tissue compartments were used to determine parameter estimates. Both the time above the MIC and 24-hour free DAL AUC/MIC were assessed as the PD target goals. The PK target for DAL was a 24-hour fAUC/MIC of 27.1 µg/mL.

**Results:**

The two dose DAL dosing regimen (1500mg x2) maintained simulated free serum concentrations above the breakpoint (0.25 ug/mL) for a median of 56 days (lower 95%CI = 33 days) and above the MIC90 (0.06 ug/mL) for > 56 days (lower 95%CI = 43 days). Six dose DAL regimen (1000mg, then 500mg weekly) maintained simulated free serum concentrations above the breakpoint for a median of > 56 days (lower 95%CI = 53 days) and above the MIC90 for > 56 days (lower 95% CI = > 56 days). Both tissue compartments in the 3 compartment PK model demonstrated very similar drug levels to serum. The lower 95^th^ percentile of 24h *f*AUC/MIC90 was maintained above 27.1 for > 56 days with both the 2-dose and 6-dose regimens.

Simulated free serum concentration of dalbavancin for modeled dosing regimens

**Figure:**
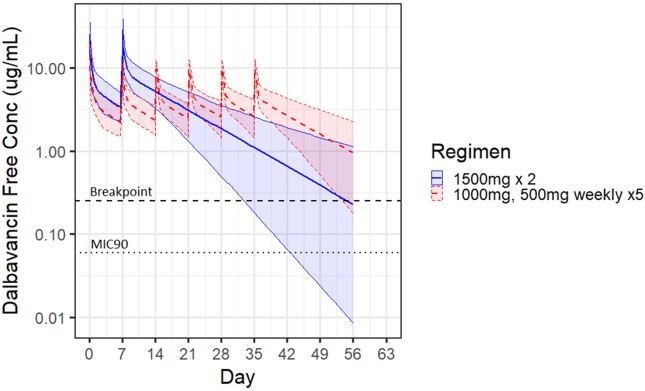

**Conclusion:**

Based on these simulations, the majority of patients maintain therapeutic DAL levels for at least 8 weeks using both modeled dosing regimens. These results, when combined with the observational data reporting clinical success, support the use of DAL when long durations of therapy are required, as for osteomyelitis or bacteremia.

**Disclosures:**

**Paul Hutson, PharmD, MS**, Revive: Grant/Research Support **Warren Rose, PharmD, MPH**, Basilea: Honoraria|Ferring: Honoraria|Merck: Grant/Research Support|Paratek: Grant/Research Support|Pfizer: Honoraria

